# Trace analysis of emerging and regulated mycotoxins in infant stool by LC-MS/MS

**DOI:** 10.1007/s00216-021-03803-9

**Published:** 2021-12-21

**Authors:** Magdaléna Krausová, Kolawole I. Ayeni, Lukas Wisgrill, Chibundu N. Ezekiel, Dominik Braun, Benedikt Warth

**Affiliations:** 1 Faculty of Chemistry, Department of Food Chemistry and Toxicology, University of Vienna, Währinger Straße 38, 1090 Vienna, Austria; 2 Department of Microbiology, Babcock University, Ilishan Remo, Ogun State Nigeria; 3 Division of Neonatology, Pediatric Intensive Care and Neuropediatrics, Comprehensive Center for Pediatrics, Department of Pediatrics and Adolescent Medicine, Medical University of Vienna, 1090 Vienna, Austria

**Keywords:** Natural food contaminants, Human biomonitoring, Infant and public health, Feces, Exposome research, Sub-Saharan Africa

## Abstract

Infants are sensitive to negative effects caused by food contaminants such as mycotoxins. To date, analytical methods assessing mycotoxin mixture exposure in infant stool are absent. Herein, we present a novel multi-mycotoxin LC-MS/MS assay capable of detecting 30+ analytes including the regulated mycotoxin classes (aflatoxins, trichothecenes, ochratoxins, zearalenone, citrinin), emerging *Alternaria* and *Fusarium* toxins, and several key metabolites. Sample preparation consisted of a ‘dilute, filter, and shoot’ approach. The method was in-house validated and demonstrated that 25 analytes fulfilled all required criteria despite the high diversity of chemical structures included. Extraction recoveries for most of the analytes were in the range of 65–114% with standard deviations below 30% and limits of detection between 0.03 and 11.3 ng/g dry weight. To prove the methods’ applicability, 22 human stool samples from premature Austrian infants (*n* = 12) and 12-month-old Nigerian infants (*n* = 10) were analyzed. The majority of the Nigerian samples were contaminated with alternariol monomethyl ether (8/10) and fumonisin B_1_ (8/10), while fumonisin B_2_ and citrinin were quantified in some samples. No mycotoxins were detected in any of the Austrian samples. The method can be used for sensitive human biomonitoring (HBM) purposes and to support exposure and, potentially, risk assessment of mycotoxins. Moreover, it allows for investigating potential associations between toxicant exposure and the infants’ developing gut microbiome.

**Graphical abstract:**

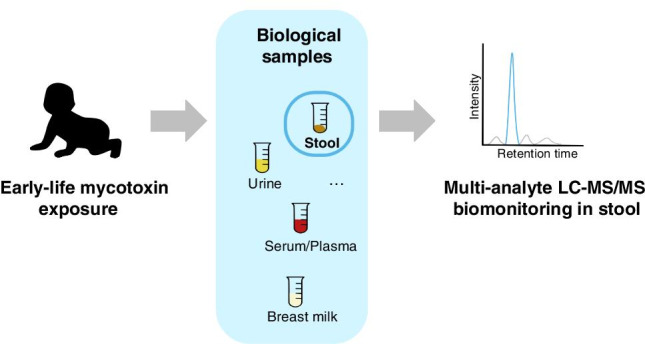

## Introduction

**Fig. 1:**
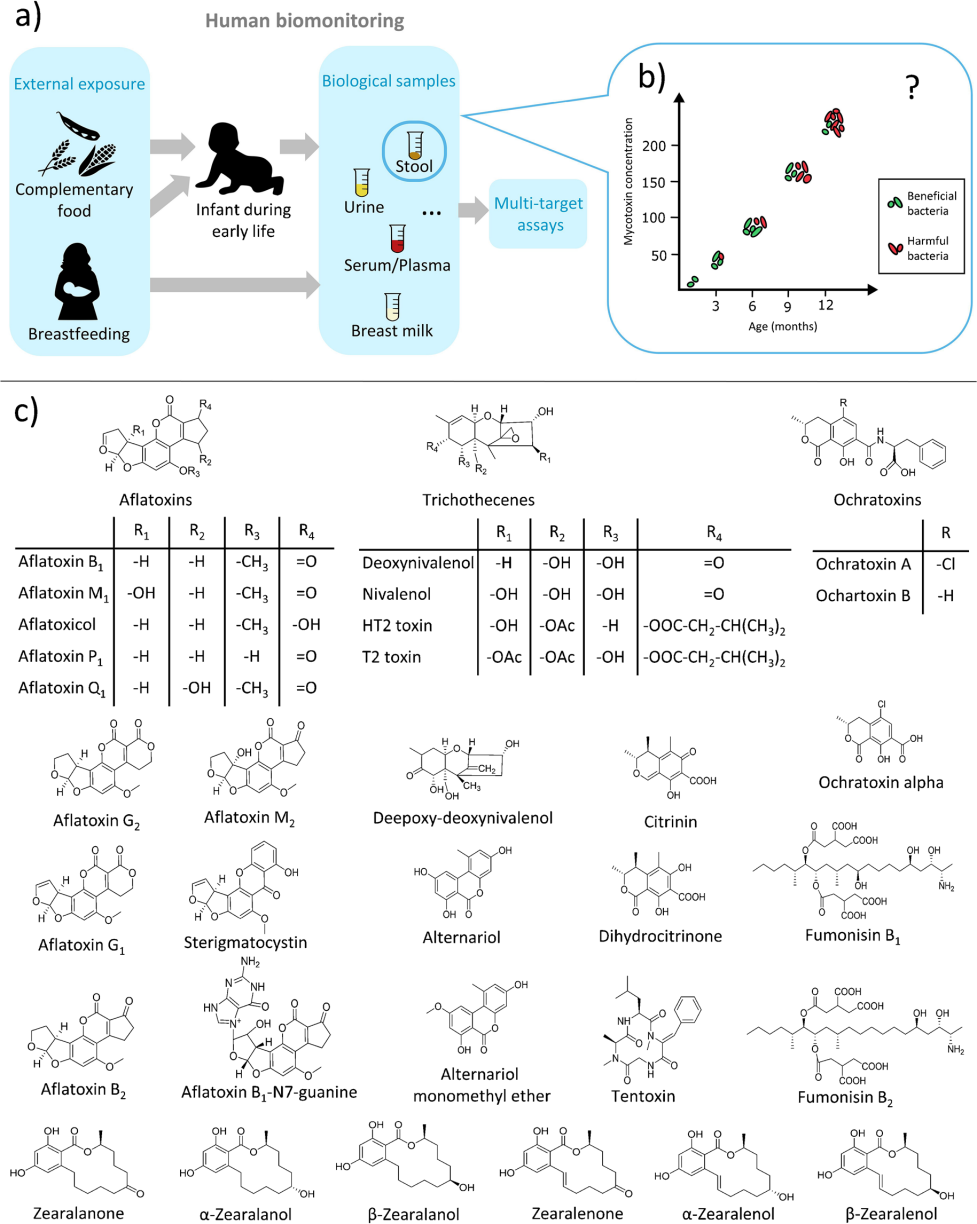
(a) Schematic illustration of the human biomonitoring (HBM) approach. An infant might be exposed to mycotoxins via complementary infant food or via breast milk. Biological samples including blood, urine, stool, breast milk, and others can be used for monitoring mycotoxin exposure using analytical methods such as LC-MS/MS. (b) Possible effects of xenobiotics such as mycotoxins on the gut microbiome development. With increasing mycotoxin concentration, the gut microbiota might become unbalanced, which could encourage colonization by more harmful bacteria. (c) Chemical structures of the 32 mycotoxins (and metabolites) that have been included in the final LC-MS/MS method

Mycotoxins are naturally occurring secondary metabolites primarily produced by toxigenic molds belonging to the *Aspergillus*, *Fusarium*, *Penicillium*, and *Alternaria* genera (Fig. 1(c)) [1]. Typically, mycotoxins contaminate food crops such as maize, rice, wheat, nuts, spices, coffee beans, and dried fruits [1, 2]. Recently, the worldwide occurrence of mycotoxins in food crops was reported to range between 60 and 80% [3]. Mycotoxin contamination of food is a global problem, but its deleterious effects are prominent in countries with high humidity and warm temperatures [4]. In humans, the main route of exposure is via ingestion of contaminated food and other possible routes include inhalation and dermal absorption [5]. Depending on the concentration level, duration, and type of mycotoxins, human exposure to mycotoxins can lead to several acute or chronic toxic effects. For example, aflatoxin B_1_ is classified as a group 1 carcinogen, which has been associated with liver cancer, whereas aflatoxin M_1_ is classified as potentially carcinogenic to humans (group 2B) [1, 6]. Fumonisins (FBs) are classified as group 2B carcinogens by the International Agency for Research on Cancer (IARC) [6] and have been implicated in poor child growth and incidence of esophageal cancer [7–9]. Ochratoxin A (OTA) and citrinin (CIT) have been shown to cause nephrotoxicity [10, 11]. Zearalenone (ZEN) can exert estrogenic effects by binding to the estrogen receptor [12, 13].

In the European Union, most major classes of mycotoxins including aflatoxins (AFs), CIT, FBs, trichothecenes, and OTA are regulated by the European Food Safety Authority (EFSA), who has set maximum levels in animal feed and food commodities for humans. However, for emerging mycotoxins, e.g., *Alternaria* mycotoxins, including alternariol (AOH), alternariol monomethyl ether (AME), and tentoxin (TEN), no guidance levels were set by EFSA due to insufficient data to assess the health risk [14]. The toxicity of and exposure to *Alternaria* toxins were recently reviewed by Aichinger et al*.* [15].

Human biomonitoring (HBM) has become an important tool for determining the internal dose of a given xenobiotic. Analysis of biological samples including breast milk, blood, saliva, stool, and urine can help to monitor levels of any xenobiotic or natural contaminants including mycotoxins (Fig. 1(a)). The biomonitoring data can be used for exposure and risk assessment and help in developing policies [16]. The preferred analytical platform for multi-analyte HBM is liquid chromatography tandem mass spectrometry (LC-MS/MS), owing to its high sensitivity, specificity, and robustness, which make it well suited for ultra-trace analysis. Developing an analytical method with the aim of including several classes of xenobiotics with different chemical properties is challenging. In general, analytical approaches taking into consideration a holistic view by analyzing numerous classes of compounds originating from different types of exposures are becoming popular [17]. Despite recent progress, there are still many knowledge gaps in areas such as mycotoxin research, where the health effects of exposure to mycotoxin mixtures or exposures during fetal and post-natal development have not been studied in detail. To date, no sensitive targeted multi-mycotoxin LC-MS/MS method for infant stool is available, to the best of our knowledge. There are a few reports where mycotoxins such as fumonisin B_1_ (FB_1_) and aflatoxin B_1_ (AFB_1_) metabolites in human stool have been investigated [18–20]. These studies, however, applied high-performance liquid chromatographic (HPLC) methods without mass spectrometric detection, and the focus was exclusively on a single mycotoxin class. Other studies that employed LC-MS-based methods targeting mycotoxins in stool are available for animals; however, like in human studies, the focus has been mostly on a single mycotoxin class instead of a comprehensive multi-class assessment [21–25]. Previously, Cao et al. developed a mass spectrometric–based method, for detecting mycotoxins in various matrices including stool, with some emerging toxins (e.g., citrinin and alternariol) not included and the samples analyzed were obtained from adults [26]. Multi-mycotoxin LC-MS-based methods are available for other frequently used HBM matrices such as urine and breast milk [27, 28]. Stool is a very important yet commonly not considered matrix in HBM, despite the fact that it is non-invasive and can provide useful exposure data. For example, analysis of the stool can give a broader insight into the level of exposure to poorly absorbed mycotoxins such as FBs, especially in high-risk mycotoxin regions. Moreover, stool reflects to a large extent the gut microbiome and it can be easily collected, compared to invasive sampling types such as biopsy or luminal brush [29]. Mycotoxins can distort the gut microbiota potentially causing poor nutrient absorption and disrupting the local immune response (Fig. 1(b)) [30]. For example, FB_1_ can induce intestinal cell apoptosis, whereas OTA was shown to decrease glucose absorption in rats [30, 31]. Alternariol has been recently reported to be absorbed by gut bacteria in vitro [32].

The main aim of this study was to develop and validate a sensitive and selective multi-mycotoxin LC-MS/MS method assessing 30+ mycotoxins/metabolites in infant stool. The performance of the method was demonstrated in a proof-of-principle study utilizing stool samples from two countries.

## Materials and methods

### Chemicals and reagents

Water (H_2_O), acetonitrile (ACN), and methanol (MeOH) (all LC-MS grade) were purchased from Honeywell (Seelze, Germany). Acetic acid (HAc) was bought from Sigma-Aldrich (Vienna, Austria). The following mycotoxin reference standards were purchased: AFB_1_, AFB_2_, AFG_1_, AFG_2_, deoxynivalenol (DON), FB_1_, FB_2_, nivalenol (NIV), OTA, sterigmatocystin (STC), T-2 toxin, alpha-zearalenol (α-ZEL), beta-zearalenol (β-ZEL), alpha-zearalanol (α-ZAL), beta-zearalanol (β-ZAL), zearalanone (ZAN), and ZEN from RomerLabs (Tulln, Austria). Enniatin A (Enn A), Enn A_1_, Enn B, Enn B_1_, and TEN were purchased from Sigma-Aldrich (Vienna, Austria). Aflatoxin metabolites AFM_1_, AFM_2_, AFP_1_, AFQ_1_, AFB_1_-N^7^-guanine (AFB-Gua) adduct, AME, AOH, beauvericin (BEA), CIT, HT-2 toxin, ochratoxin alpha (OTα), and ochratoxin B (OTB) were bought from Toronto Research Chemicals (Ontario, Canada). Dihydrocitrinone (DH-CIT) was kindly provided by Michael Sulyok (IFA-Tulln, Austria).

Solid reference standards were dissolved in ACN, except the fumonisins (ACN/H_2_O, 1/1, v/v) and AFB-Gua (ACN/H_2_O/acetic acid, 75/24/1, v/v/v), to reach individual stock solutions with final concentrations of 5–500 μg/mL which were stored at – 20 °C. Internal standards (IS) [^13^C]-AFM_1_, [^13^C]-CIT, [^13^C]-DON, [^13^C]-FB_1_, [^13^C]-NIV, [^13^C]-OTA, and [^13^C]-ZEN were purchased from RomerLabs (Tulln, Austria). [^2^H]-AOH was kindly provided by Michael Rychlik (TU Munich, Germany).

Individual stock solutions were diluted in ACN to prepare a multi-standard working solution containing all analytes in a concentration range of 3.7–16,000 ng/mL. Similarly, the IS mixture was prepared from individual stock solutions, containing the following final concentrations: [^2^H]-AOH (1.5 ng/mL), [^13^C]-AFM_1_ (0.3 ng/mL), [^13^C]-CIT (0.3 ng/mL), [^13^C]-DON (3 ng/mL), [^13^C]-FB_1_ (50 ng/mL), [^13^C]-NIV (3 ng/mL), [^13^C]-OTA (3 ng/mL), and [^13^C]-ZEN (0.3 ng/mL).

### Sample collection

Stool samples (*n* = 12) from 8 infants that were analyzed individually (for exposure assessment) but also pooled (for method development and in-house validation) were obtained from 6- to 8-week-old extremely premature infants (< 28^th^ week of gestational age and < 1000 g birth weight) in 2021 in Vienna, Austria. All infants were fully enteral fed and exclusively received own mothers’ milk or pooled pasteurized human breast milk with 4% of a bovine fortifier (Beba FM85, Nestle). The study was approved by the local ethics committee of the Medical University of Vienna (1348/2017).

For the Nigerian proof-of-principle study, stool samples (*n* = 10) were collected from 12-month-old infants from Ogun state (5 males, 5 females). These samples are part of an ongoing, larger longitudinal study (2019–2021) that aims to correlate toxicant exposure with effects on the gut microbiome. Infants consumed mothers’ breast milk and complementary food mostly consisting of cereals, infant formula, nuts, and tuber. The weight and the height of the children were taken using a digital scale and measuring tape, respectively. The stool samples were collected by mothers or by trained study personnel directly from diapers into falcon tubes (50 mL) and stored immediately at – 20 °C. The samples were transported on dry ice for LC-MS/MS analysis. Ethical approval was obtained from the Ethical Committee of Babcock University (BUHREC585/18, BUHREC421/21R). For both studies, all parents gave written informed consent prior to study inclusion.

### Sample preparation protocol

**Fig. 2:**
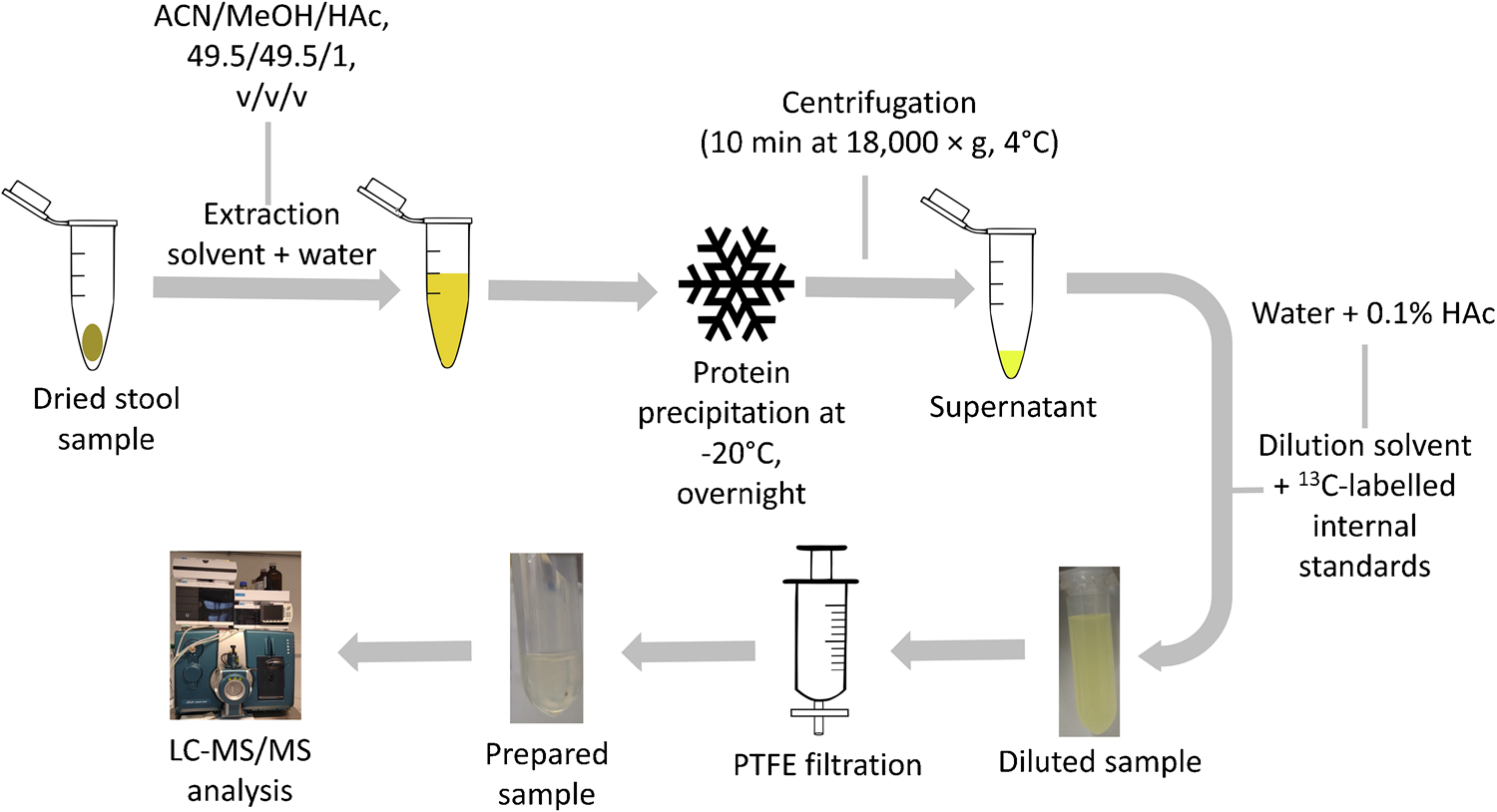
Schematic illustration of the sample preparation procedure. After drying the stool sample, extraction solvent is added followed by a protein precipitation step. The sample is then centrifuged, and the supernatant is further diluted and filtered through a PTFE filter into an LC glass vial. Finally, the diluted and filtered sample is injected onto an LC-MS instrument

During method development, different clean-up protocols were tested and optimized. As the final extraction method, samples were diluted and filtered using polytetrafluoroethylene (PTFE) filters (pore size 0.20 µm, diaphragm diameter 13 mm, Macherey-Nagel, Germany), as summarized in Fig. 2. In brief, wet premature infant stool (*n* = 12) was pooled together and homogenized with a spatula. Approximately 40 mg of the pooled sample was weighed in micro reaction tubes (2 mL) followed by a drying step (24 h) in a vacuum concentrator (Labconco, MO, USA). After drying, the weight of the samples was recorded again (on average approx. 30% of the original weight; range 26–34%). Subsequently, water was added (40 µL per 20 mg) followed by vortexing. The extraction solvent (ACN/MeOH/HAc, 49.5/49.5/1, v/v/v) was added (160 µL per 20 mg) followed by thorough vortexing and ultrasonication in an ice bath (15 min). The samples were stored overnight at − 20 °C to precipitate the proteins, followed by centrifugation (10 min at 18,000 × g, 4 °C), after which the supernatants were transferred to new reaction tubes. The supernatants were diluted (1:10) with water containing 0.1% HAc and 10 µL IS mixture. The final concentration of the individual IS in the samples was as follows: [^2^H]-AOH (0.075 ng/mL), [^13^C]-AFM_1_ (0.015 ng/mL), [^13^C]-CIT (0.015 ng/mL), [^13^C]-DON (0.15 ng/mL), [^13^C]-FB_1_ (2.5 ng/mL), [^13^C]-NIV (0.15 ng/mL), [^13^C]-OTA (0.15 ng/mL), and [^13^C]-ZEN (0.015 ng/mL). Then, the diluted samples were filtered through PTFE filters. The resulting overall dilution factor of this procedure was 1:100. The filtrates were transferred to amber-LC vials with micro-inserts. A volume of 5 µL were injected into the LC-MS/MS system.

### LC-MS/MS parameters and analysis

The analysis was performed using an Agilent 1290 Infinity II LC coupled to a Sciex QTrap 6500^+^ (Darmstadt, Germany) mass spectrometer (MS), which was equipped with a Turbo-V™ electrospray ionization (ESI) source. The separation was achieved on an Acquity HSS T3 column (2.1 × 100 mm, 1.8 μm particle size) purchased from Waters (Vienna, Austria), protected with a VanGuard pre-column (1.8 μm, Waters, Vienna, Austria). The mobile phase was composed of an aqueous solvent A (H_2_O + 0.1% HAc) and solvent B (MeOH + 0.1% HAc). The gradient was based on the method developed previously by Braun et al. [28]. In brief, the gradient was as follows: the first 0.5 min, the content of B was kept at 10%. From 0.5 to 1 min, the eluent B was increased to 35% and then to 60% at min 3. The eluent B was then further increased to reach 97% at min 10 and kept constant at these conditions for 6 min. Eluent B was then rapidly dropped to the starting conditions and the column was re-equilibrated for 2.9 min before the next injection. The overall run time was 19 min. The column oven was maintained at 40 °C and the autosampler at 7 °C. The measurements were done in scheduled multiple reaction monitoring (MRM) mode, and the compounds were ionized in either positive or negative ESI mode using fast polarity switching. The settings of the ion source were as follows: source temperature 450 °C, curtain gas 30 psi, collision gas high, ion source gasses (sheath and drying gas) 80 psi and the ion spray voltage was set to 4500 V in positive and − 4500 V in negative mode. Complete MS parameters for all the analytes are summarized in Table 1.

**Table 1:** MS and MS/MS parameters including the retention time, precursor and product ion (*m*/*z*), ion species, de-clustering potential (DP), collision energy (CE), cell exit potential (CXP), and ion ratio (%)

**Analyte**	**Retention time**	**Precursor ion**	**Product ion**	**Ion species**	**DP**	**CE**	**CXP**	**Ion ratio**
	(min)	(*m*/*z*)	(*m*/*z*)		(V)	(V)	(V)	(%)
**Aflatoxicol**	5.9	297.0	269.1/115.0	[M−H_2_O+H]^+^	71	29/83	12/14	78
**Aflatoxin B**_**1**_	5.2	313.0	241.0/213.0	[M+H]^+^	106	49/61	14/16	67
**Aflatoxin B**_**2**_	5.0	315.0	259.2/243.0/203.0	[M+H]^+^	96	43/53/49	18/16/12	44
**Aflatoxin G**_**1**_	4.7	329.1	243.1/200.0	[M+H]^+^	86	39/59	14/12	65
**Aflatoxin G**_**2**_	4.5	331.1	313.2/245.2	[M+H]^+^	111	35/43	18/14	55
**Aflatoxin M**_**1**_	4.5	329.1	273.2/229.1	[M+H]^+^	91	35/59	16/12	50
^**13**^**C-Aflatoxin M**_**1**_	4.5	346.0	288.2	[M+H]^+^	91	35	16	–
**Aflatoxin M**_**2**_	4.3	331.0	285.2/259.0/241.0	[M+H]^+^	96	33/33/57	14/16/14	78
**Aflatoxin P**_**1**_	4.8	299.1	270.7/215.1/171.1	[M+H]^+^	126	35/38/56	18/11/17	31
**Aflatoxin Q**_**1**_	4.4	328.7	206.0/177.0	[M+H]^+^	121	33/47	14/12	73
**Aflatoxin B**_**1**_**-N7-guanine**	4.0	480.0	152.1/135.0	[M+H]^+^	46	23/85	10/14	39
**Alternariol**	6.4	257.0	215.0/213.0	[M−H]^−^	− 100	− 36/− 34	− 11/− 11	64
^**2**^**H-Alternariol**	6.4	261.0	150.0	[M−H]^−^	− 110	− 46	− 5	–
**Alternariol monomethyl ether**	8.2	271.1	256.0/227.0	[M−H]^−^	− 95	− 32/− 50	− 13/− 9	19
**Citrinin**	6.2	281.0	249.0/205.0	[M+MeOH−H]^−^	− 50	− 24/− 33	− 7/− 7	56
^**13**^**C-Citrinin**	6.2	294.3	217.1	[M+MeOH−H]^−^	− 40	− 32	− 17	–
**Deepoxy-deoxynivalenol**	3.8	339.1	248.9/59.1	[M+OAc]^−^	− 70	− 18/− 20	− 17/− 9	17
**Deoxynivalenol**	3.2	355.1	59.2/265.2	[M+OAc]^−^	− 70	− 40/− 24	− 8/− 13	28
^**13**^**C-Deoxynivalenol**	3.2	370.1	278.8	[M+OAc]^−^	− 20	− 22	− 15	–
**Dihydrocitrinone**	5.2	265.0	177.0/203.0/147.1	[M−H]^−^	− 25	− 34/− 40/− 46	− 11/− 17/− 15	22
**Fumonisin B**_**1**_	5.8	722.5	334.4/352.3	[M+H]^+^	121	57/55	4/12	103
^**13**^**C-Fumonisin B**_**1**_	5.8	756.3	356.3	[M+H]^+^	130	46	10	–
**Fumonisin B**_**2**_	7.2	706.5	336.4/318.4	[M+H]^+^	126	59/51	8/2	53
**HT-2 toxin**	6.2	442.2	263.1/215.0	[M+NH_4_]^+^	76	21/21	19/19	55
**Nivalenol**	2.8	371.1	281.1/59.1	[M+OAc]^−^	− 75	− 22/− 42	− 15/− 7	84
^**13**^**C-Nivalenol**	2.8	386.0	295.2	[M+OAc]^−^	− 75	− 22	− 15	–
**Ochratoxin A**	7.7	404.0	239.0/102.0	[M+H]^+^	91	37/105	16/14	36
^**13**^**C-Ochratoxin A**	7.7	424.0	250.0	[M+H]^+^	51	33	12	–
**Ochratoxin B**	6.6	370.1	205.0/103.1	[M+H]^+^	86	33/77	12/16	34
**Ochratoxin α**	5.1	254.9	166.9/123.0/110.9	[M−H]^−^	− 90	− 36/− 40/− 44	− 11/− 17/− 21	21
**Sterigmatocystin**	8.1	325.1	281.1/310.2	[M+H]^+^	96	51/35	16/18	83
**T-2 toxin**	7.0	467.3	215.2/185.1	[M+NH_4_]^+^	56	29/31	18/11	84
**Tentoxin**	6.5	413.3	141.0/271.1	[M−H]^−^	− 105	− 30/− 24	− 11/− 15	55
**Zearalanone**	7.5	319.1	107.0/137.0	[M−H]^−^	− 145	− 40/− 38	− 13/− 17	57
**α-Zearalanol**	7.2	321.1	277.1/235.1/161.0	[M−H]^−^	− 120	− 30/− 32/− 38	− 18/− 17/− 9	8
**β-Zearalanol**	6.4	321.1	277.05/303.05	[M−H]^−^	− 120	− 30/− 30	− 18/− 20	29
**Zearalenone**	7.7	317.1	175.0/131.1/160.0	[M−H]^−^	− 110	− 34/− 42/− 40	− 13/− 8/− 11	35
^**13**^**C-Zearalenone**	7.7	335.2	185.1	[M−H]^−^	− 110	− 34	− 13	–
**α-Zearalenol**	7.4	319.2	160.1/130.1	[M−H]^−^	− 115	− 44/− 50	− 13/− 20	69
**β-Zearalenol**	6.7	319.2	160.0/130.0	[M−H]^−^	− 115	− 44/− 50	− 13/− 20	67

For data acquisition, the Analyst (version 1.7.1) software was applied, and for quantification, SCIEX OS (version 1.6 and 2.0) was used. A system quality control (QC) sample was measured before and after each measurement sequence (in triplicates) to ensure acceptable performance of the instrument. Additional QC samples were included for the Nigerian measurements: the dried stool samples were spiked at two levels (low and high) before extraction (triplicates for each level) with a mycotoxin mixture and were left to dry (with open lids) for approximately 45 min. The spiking levels were selected based on preliminary measurements, and the spiking levels were aimed for 5× and 15× the respectively LOQ values. The extraction was carried out as described in the sample preparation protocol. The quantitative results were required to be < 20% RSD for all analytes.

### In-house validation

In-house validation was performed according to the EuraChem Laboratory Guide and the European Commission decision 2002/657/EC concerning the performance of analytical methods and the interpretation of results [33, 34]. The following parameters were considered: linearity, extraction recovery (*R*_E_), repeatability (intraday precision, RSD_r_), intermediate precision (interday precision, RSD_R_), selectivity, signal suppression or enhancement (SSE), and sensitivity. A pooled stool derived from 8 newborns was used as matrix blank as no commercial reference material was available. Calibration standards were prepared in dilution solvent (H_2_O + 0.1% HAc) and pooled blank matrix, each consisting of five or six calibration points ranging from 0.00025 to 304.5 ng/mL. The highest standard was prepared by diluting the multi-analyte working solution by 1:10 (v/v) in blank matrix or in dilution solvent (H_2_O + 0.1% HAc). The standards of lower concentrations were prepared by serial dilution, using the highest standard. A weighing factor of 1/*x* was used for both calibration curves, which was generated by plotting the measured concentrations against the peak area of the respective analyte. The peak area ratios were used for the calculations of analytes with available IS; for the other analytes, solely the peak area was used. Spiked samples, matrix-matched standards, and solvent standards were measured in three distinct sequences over a period of 10 weeks. The linearity was evaluated by considering the regression coefficient *R*^2^ of the matrix-matched calibration curve for all analytes. The recovery experiments were done by spiking blank matrix samples before the extraction procedure at two different concentration levels, each in triplicate. The *R*_E_ was calculated using the linear equation of the matrix-matched calibration standards. The calculated concentration of spiked samples was divided by the theoretical spiking concentration and reported in percent (%) for the respective analyte. The percentage of repeatability (RSD_r_) and intermediate precision (RSD_R_) were each calculated by using the standard deviation of the average concentration determined in spiked samples divided by the average concentration determined in spiked samples and multiplied with 100. The number of replicates was 6 and 9 for RSD_r_ and RSD_R_, respectively. The selectivity was determined by comparing the blank samples with the spiked samples and by investigating the monitored transitions during proof-of-principle experiments.

Positive identification was based on four factors including matching retention time, the presence of quantifier and qualifier ions and their matching ion ratios. The ion ratios (in %) were evaluated from matrix calibration (quantifier’s calculated concentration divided by qualifier and multiplied by 100). The matrix effect (in %) was assessed using the SSE, which was calculated by dividing the slope of the matrix-matched calibration curve by the slope of the solvent calibration curve and multiplying by a factor of 100. The limit of detection (LOD) and quantification (LOQ) values were determined by calculating the average concentration of the lowest quantifiable signal of the respective analyte and dividing this value by the average signal-to-noise (*S*/*N*) ratio (generated by algorithm in SCIEX OS). Resulting values were multiplied by a factor of three and six to derive the instrumental LOD and LOQ values, respectively, which were finally converted to nanograms per gram of dried stool. All quantitative results in naturally contaminated samples were corrected for the extraction efficiency and the weight (expressed in the results as ng/g dry weight).

## Results and discussion

### Optimization of the sample preparation protocol

Herein, we present the development of the first selective and sensitive LC-MS/MS method targeting multiple mycotoxin classes in infant stool. The method includes 32 mycotoxins including some of their metabolites, out of which 25 met all the requirements laid out in the validation guidelines. The results of the validation experiments are summarized in Table 2.

**Table 2:** In-house validation results of the method including spiking levels, extraction efficiency (*R*_E_), intermediate precision (RSD_R_), repeatability (RSD_r_), signal suppression/enhancement (SSE), limits of detection (LOD), and limits of quantification (LOQ)

**Analyte**	**Spiking level**^a^	***R***_**E**_** ±RSD**_**R**_ **Low level**	***R***_**E**_** ±RSD**_**R**_ **High level**	**RSD**_**r**_^b^	**SSE**	**LOD**	**LOQ**
	**(ng/g)**	**(%)**	**(%)**	**(%)**	**(%)**	**(ng/g)**	**(ng/g)**
**Aflatoxicol**	21/63	80 ± 32	80 ± 15	5/4	93	0.8	1.6
**Aflatoxin B**_**1**_	3.5/10	84 ± 22	79 ± 21	18/18	84	0.2	0.4
**Aflatoxin B**_**2**_	3/9	83 ± 28	86 ± 15	36/13	85	0.3	0.6
**Aflatoxin G**_**1**_	5/15	73 ± 12	80 ± 14	14/3	96	0.5	1.0
**Aflatoxin G**_**2**_	15/45	80 ± 22	88 ± 13	17/7	98	1.2	2.4
**Aflatoxin M**_**1**_	5/15	91 ± 18	94 ± 11	9/7	102	0.2	0.4
**Aflatoxin M**_**2**_	15/45	61 ± 35	80 ± 14	15/11	102	1.0	2.0
**Aflatoxin P**_**1**_	30/90	96 ± 19	93 ± 14	10/11	86	2.2	4.4
**Aflatoxin Q**_**1**_	5/15	79 ± 21	82 ± 18	34/6	96	0.5	1.0
**Aflatoxin B**_**1**_**-N7-guanine**	4/12	84 ± 17	87 ± 12	7/4	110	0.1	0.2
**Alternariol**	10/30	68 ± 28^c^	78 ± 8	40^c^/9	96	0.8	1.6
**Alternariol monomethyl ether**	0.7/2.2	40 ± 27	49 ± 16	23/7	83	0.03	0.06
**Citrinin**	1.5/4.5	–	51 ± 25	–/22	123	0.04	0.08
**Deepoxy-deoxynivalenol**	225/675	94 ± 14	98 ± 13	5/1	45	1.4	2.4
**Deoxynivalenol**	80/240	111 ± 13	96 ± 12	22/13	54	6.3	12.6
**Dihydrocitrinone**	18/53	87 ± 21	91 ± 14	8/9	131	1.4	2.8
**Fumonisin B**_**1**_	35/105	140 ± 39^d^	164 ± 45	–^d^/23	141	4.9	9.8
**Fumonisin B**_**2**_	18/83	94 ± 72^e^	65 ± 26	13/14	212	1.3	2.6
**HT-2 toxin**	1015/3045	81 ± 12	95 ± 9	14/9	99	92.4	184.8
**Nivalenol**	31/93	–	97 ± 29	–/7	29	11.3	22.6
**Ochratoxin A**	5/15	132 ± 28	105 ± 33	8/9	112	0.4	0.8
**Ochratoxin B**	3/9	114 ± 29	106 ± 17	8/5	113	0.1	0.2
**Ochratoxin α**	35/105	97 ± 24	94 ± 11	7/4	108	2.9	5.8
**Sterigmatocystin**	0.5/1.5	24 ± 32	29 ± 15	20/11	97	0.03	0.06
**T-2 toxin**	160/480	–	99 ± 13	–/10	105	74.1	148.2
**Tentoxin**	5/15	75 ± 13	81 ± 12	6/3	83	0.2	0.4
**Zearalanone**	25/75	56 ± 12	54 ± 12	12/5	83	0.8	1.6
**α-Zearalanol**	15/45	68 ± 10	67 ± 4	3/2	88	0.4	0.8
**β-Zearalanol**	15/45	78 ± 10	80 ± 6	9/1	88	0.6	1.2
**Zearalenone**	7.5/22	100 ± 62	72 ± 39	37/21	71	0.4	0.8
**α-Zearalenol**	7/21	60 ± 22	57 ± 8	8/1	78	0.3	0.6
**β-Zearalenol**	15/45	76 ± 14	75 ± 13	6/5	81	0.2	0.4

^a^Spiking levels in order: low, high

^b^Intraday precision RSD in order: low spiking level, high spiking level

^c^Based on 11/12 samples

^d^Based on 7/12 samples, it was not possible to calculate RSD_r_

^e^Based on 10/12 samples

The aim of the sample preparation was to develop a fast and simple clean-up procedure. As there are no available simple clean-up methods for infant stool published in the literature, animal studies were used instead. The initial sample preparation conditions were based on a rat study by Puntscher et al. [35], where the samples were extracted followed by an overnight protein precipitation step and centrifugation. The samples were then evaporated in a vacuum concentrator, reconstituted, and analyzed. This “evaporation method” was tested and for comparison purpose, a simple “extract-dilute-shoot” approach (dilution 1:10) was explored additionally. The evaporation step was omitted after the first tests as the resulting pellet was difficult to re-dissolve. The “extract-dilute-shoot” approach resulted in higher recoveries compared to the “evaporation method” and thus was further optimized. Several dilution factors were tested but dilution below a factor of 100 showed worse results due to concentrated and crude extracts. In addition, a solid phase extraction step (SPE) and filtration through PTFE filters were tested to increase sensitivity and purify sample extracts. The results from the SPE showed that several analytes were lost during the procedure and the extraction recoveries were much lower in comparison to the filtered samples. The extraction followed by dilution and filtration proved to be the most appropriate clean-up approach, with highest extraction recoveries and acceptable LOD and SSE values. This method was chosen for the subsequent in-house method validation with a final dilution factor of 100. The chosen dilution factor was a compromise to achieve sufficient sensitivity and “clean” samples to avoid severe contamination of the LC-MS instrument.

### In-house validation and quality control measures

In-house method validation was performed according to EuraChem and the European Commission Decision 2002/657/EC [33, 34]. Results are reported in Table 2. Overall, for 25 mycotoxins, all parameters were fully in agreement with the requirements. The remaining mycotoxins which did not meet all the criteria for both spiked concentrations included FB_1_, STC, OTA, and AME due to issues with the extraction recovery. For the remaining NIV, CIT, and T2-toxin, only the high spiking level was evaluated as at the lower level the peak intensities were below the LOQ.

Premature infant stool samples from Austria were used for the method validation measurements. Pre-experiments showed that the analysis of each individual sample (*n* = 12) had no detectable levels of mycotoxins. Thus, individual samples were pooled and used as a blank matrix due to the current lack of suitable reference material. The estimated LOD and LOQ values for 20 analytes were below 1 ng/g and 2 ng/g, respectively. The highest sensitivity was observed for *Alternaria* toxins, zearalenone with derivatives, and aflatoxins. In general, the polar trichothecenes had higher LODs (> 1 ng/g), in particular T-2 (LOD of 74 ng/g) and HT-2 (LOD of 92 ng/g) toxins. Other analytes with higher LOD values included NIV (11 ng/g), DON (6.3 ng/g), and FB_1_ (4.9 ng/g). The *R*_E_ ranged from 54 to 114% for the fully validated analytes and were in agreement with respective guidelines. The mycotoxins which had an *R*_E_ below 50% or above 120% included STC (24% and 29%), AME (40% and 49%), FB_1_ (140% and 164%), and OTA (132%). In addition, all the ENNs and BEA could not be recovered due to the filtration step; hence, these were excluded from the final method. A possible explanation is that the stool matrix is an issue during filtration, since the same filter type was used previously to effectively recover the same analytes in breast milk [28].

RSD_R_ was ranging from 4 to 35% for most of the analytes, and the RSD_r_ was similar with a range of 1–40% for all the mycotoxins. The RSD_r_ was slightly higher than the intermediate precision, which could be due to the impact of the matrix on the column. The analytes which had a RSD_R_ higher than 35% included FBs and ZEN. Very high RSD_R_s for FB_1_ (39% and 45%) and FB_2_ (72%) were observed which could be due to the lower sample number that was used for recovery calculations or due to the specific chemical properties of the fumonisins. The high RSDs for FBs and ZEN should be further investigated in the future. The RSD values are in general high, but based on the complexity of the matrix and the simple clean-up procedure, we expected higher variances. Linearity was evaluated by using the *R*^2^ of matrix-matched calibration samples, and for all analytes, this value was above 0.98. The SSE was assessed by a comparison of the slopes of the solvent and the matrix-matched calibration curve and calculating an average. Overall, SSE was between 80 and 120% for most analytes; however, strong signal enhancement was observed for FB_1_ and FB_2_ (141% and 212%), respectively. In contrast, signal suppression was noted for NIV, DON, and DOM-1 (29%, 54%, and 45%), respectively. Signal suppression was also observed for ZEN (71%) and α-ZEL (78%); however, these were in the expected range for zearalenone and its derivatives. Although seven analytes did not fulfill all validation criteria, they could still be used for screening as was done in the Nigerian pilot study. Importantly, internal standards were utilized when available ([^2^H]-AOH, [^13^C]-AFM_1_, [^13^C]-CIT, [^13^C]-DON, [^13^C]-FB_1_, [^13^C]-NIV, [^13^C]-OTA, and [^13^C]-ZEN), accounting for the filtration step during sample preparation. The internal standards could not account for the whole extraction procedure as the required concentrations would have been rather high and cost intensive. However, adding them before extraction in future applications seems to be required, at least for some analytes. QC samples to be applied in future epidemiological cohort studies will be prepared by using a pooled matrix blank (Austrian samples used in the method validation), which was demonstrated not to be contaminated and can be used as a material for comparing performance in-house. These samples can be spiked and subsequently extracted in the future in the same way as during method validation, with a higher spiking level to ensure proper extraction and sample preparation.

The limitations of the current method are relatively low extraction recoveries and high RSDs for several analytes which are likely caused by the highly complex matrix. Probably the biggest limitation is the dilution factor of 100 used during the sample preparation, which was a compromise between sensitivity, matrix effects, instrument pollution, and sample throughput. However, the resulting LOD values were relatively low considering the high dilution factor.

### Application to biological samples

**Fig. 3:**
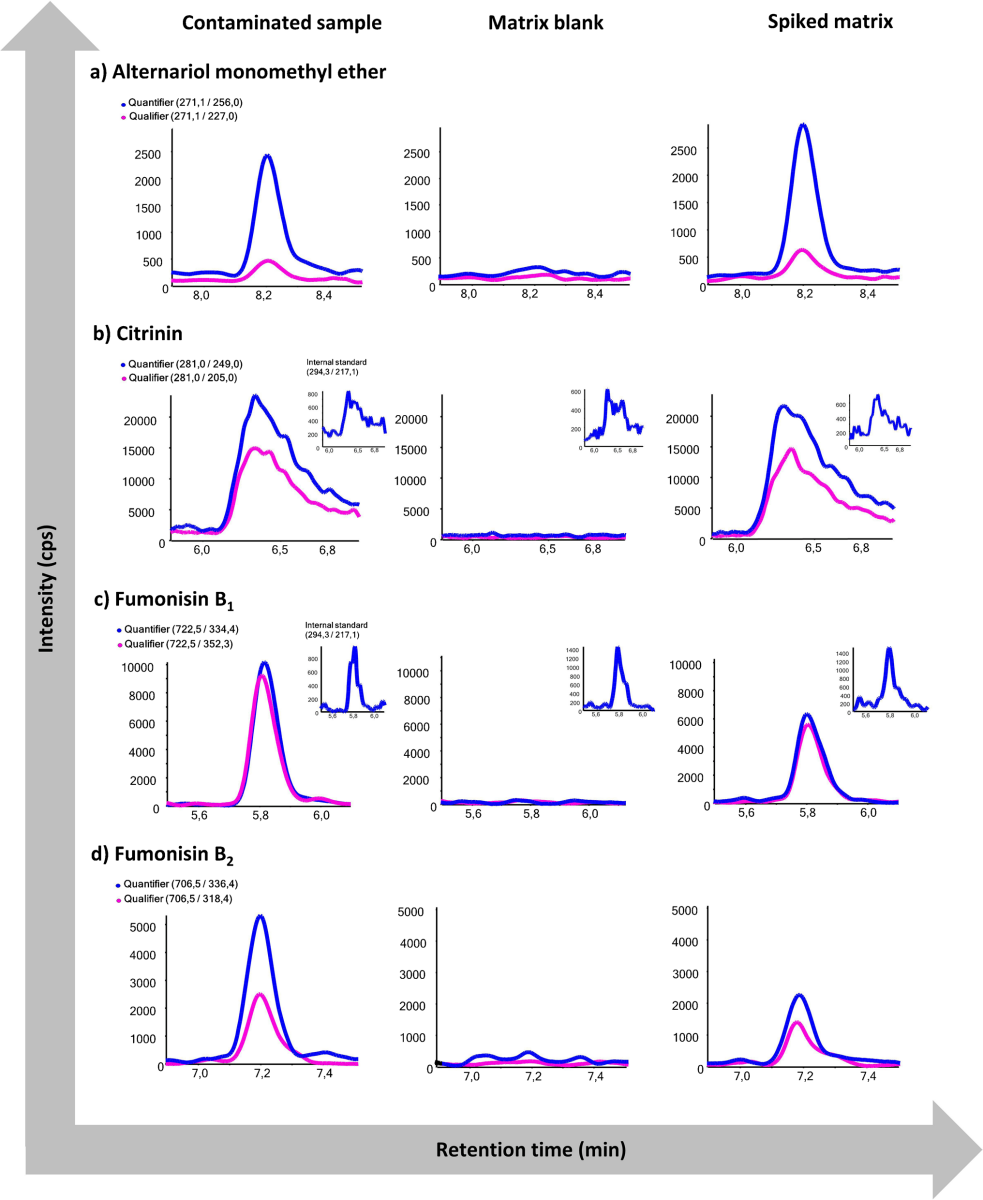
MRM-chromatograms of (a) alternariol monomethyl ether (AME), (b) citrinin (CIT), (c) fumonisin B_1_ (FB_1_), and (d) fumonisin B_2_ (FB_2_), comparing a naturally contaminated infant stool sample from the Nigerian cohort, the matrix blank of pooled Austrian infant stool samples, and the spiked matrix blank. For FB_1_ and CIT, ^13^C-labeled internal reference standards were utilized. Concentrations in the contaminated Nigerian samples were determined to be 4 ng/g (AME), 64 ng/g (CIT), 76 ng/g (FB_1_), and 339 ng/g (FB_2_)

Analysis of the stool samples from Austrian premature infants with the established method revealed no contamination; thus, the sample pool was considered as matrix blank and used in further measurements. As a proof of principle, the method was applied to a subset of Nigerian samples (*n* = 10), which were part of a larger ongoing study to be published elsewhere. The average weight (kg) and height (cm) of the females (*n* = 3) were above the 15^th^ and around the 85^th^ percentile of child growth standards, respectively. In contrast, the average weight (kg) and height (cm) of the males (*n* = 4) were around the 50^th^ and above the 15^th^ percentile of growth standards, respectively [36]. For the rest of the participants (2 females and 1 male), the weight and height were not recorded. Overall, all the samples were contaminated either with FB_1_ (8/10), FB_2_ (5/10), AME (8/10), or CIT (3/10) (Table 3). Representative MRM chromatograms of contaminated samples are shown in Fig. 3. No other mycotoxins including aflatoxins, ochratoxins, trichothecenes, or zearalenone were detected in the samples. All of the samples contaminated with FB_1_ were also contaminated with FB_2_ at concentrations ranging from 20 to 471 ng/g and 18 to 883 ng/g, respectively. Most of the positive samples were contaminated with several mycotoxins at the same time. Two samples (2 and 7) were contaminated with two mycotoxins (FB_1_ and AME), three samples (6, 8, and 9) were contaminated with three mycotoxins (FB_1_, FB_2_, and AME or CIT), and samples 4 and 5 were contaminated with all four mycotoxins.

**Table 3:** Results of the Nigerian proof-of-principle experiment demonstrating the applicability of the LC-MS/MS method in a small set of Nigerian infant stool samples. Results are reported in nanograms per gram dried stool. Results for CIT and FB_1_ were corrected by their respective internal standard

	**Alternariol monomethyl ether (ng/g)**	**Citrinin (ng/g)**	**Fumonisin B**_**1**_ **(ng/g)**	**Fumonisin B**_**2**_ **(ng/g)**
**Sample**	**(ng/g)**			
1	10	< LOD	< LOD	< LOD
2	29	< LOD	45	< LOD
3	16	< LOD	< LOD	< LOD
4	2	44	86	18
5	4	64	76	339
6	6	< LOD	355	222
7	10	< LOD	103	< LOD
8	10	< LOD	471	258
9	< LOD	104	321	883
10	< LOD	< LOD	20	< LOD

Contamination of samples was expected, because exposure to mycotoxins is a common problem in Nigeria [37, 38] and several mycotoxins including AFs, FB_1_, OTA, and AME were detected in either breast milk of Nigerian mothers or urine of infants [28, 39, 40]. However, higher contamination levels were reported in complementary food [41, 42]. Hence, the results from the present study suggest that the infants were exposed to mycotoxins mainly via complementary food. Compared to exclusively breastfed infants, non-breastfed infants or infants being breastfed and consuming other baby food are at higher risk of exposure to mycotoxins as shown by the recent study of Ezekiel et al. [43].

The novel method developed herein can be used as a foundation to comprehensively explore the exposure of infants to mycotoxins. It is worth noting that the *Alternaria* mycotoxin AME, which is not regulated in the European Union, was quantified in most of the samples, suggesting that it can be commonly found, at least in Ogun state, southwest Nigeria. A recent surveillance study from this state revealed AME in industrially processed fruit juice consumed by children albeit in very low concentrations [44]. Thus, more attention should be paid to AME and other related mycotoxins. Results for FB_1_ and CIT also call for concern as it was shown previously that complementary food fed to infants and young children is frequently contaminated with these two mycotoxins [41, 42, 45, 46]. In general, as described above, complementary food, in contrast to breast milk, was shown to be contaminated at higher levels and by more diverse mycotoxin classes [47]. In general, the reasons for frequent mycotoxin contamination in developing countries include poor agricultural and storage practices [48, 49]. Hence, it is likely that exclusively breastfed infants would have less contaminated stool. According to the National Bureau of Statistics and United Nations Children’s Fund, only about 24% of infants are exclusively breastfed from birth to 6 months [50]. This statistic suggests that exclusive breastfeeding is not common in Nigeria. Nevertheless, one of the most straightforward and cheapest approaches to minimize exposure to mycotoxins would be to prolong breastfeeding for at least 6 months as recommended by the World Health Organization (WHO) [51]. This not only would lead to lower natural contaminant exposure but, in addition, is known to positively affect mother-child bonding and the health of both individuals [52, 53]. It is very important to encourage and educate the general population about the clear benefits of breastfeeding, especially in economically less developed countries [54].

A comparison of the presented results with those of previously published studies revealed expected differences and a few similarities. For example, in the paper by Chelule et al. [19], in which FB_1_ was investigated, the estimated LOD was 50 ng/g in comparison to 5 ng/g in this study. The range of FB_1_ quantified in the stool samples from households in rural and urban areas in South Africa (KwaZulu Natal area) was 0.5–39 mg/kg [19], which is very high in comparison with the results reported here (max 471 ng/g). In another study by Phoku et al., the FB_1_ levels ranged from 0.3 to 464 ng/g in stool samples from Limpopo province in South Africa [20], which is similar to the FB_1_ levels detected in the Nigerian samples. In the study of Cao et al. [26], an LC-MS/MS method for adult stool was developed including mycotoxins such as fumonisins, aflatoxins, zearalenones, ochratoxin, and deoxynivalenol. The LOQs reported were lower than the LOQs in our method. For instance, the LOQs reported for FB_1_ and AFM_1_ were 0.5 ng/g and 0.2 ng/g, respectively [26], compared to 9.8 ng/g and 0.4 ng/g reported in the present study. The study of Cao et al. [26], however, did not include mycotoxins such as CIT or AME, which were covered using our method. In addition, the method was developed using adult stool, whereas the focus of this study is on premature infants and 12-month-old infants. In the three samples that were analyzed, AFB_1_, FB_1_, OTA, α-ZEL, β-ZEL, neosolaniol, and T-2 triol were detected.

In summary, we have developed the first multi-mycotoxin LC-MS/MS method for human infant stool. The major advantage of the method is the straightforward clean-up of the samples which only includes extraction, dilution, and filtration steps; thus, potential analyte losses during preparation are minimized. Another main advantage is that the method includes several major mycotoxin classes with different chemical properties. In the future, the method can be used for exposure assessments and screening of highly exposed populations in regions such as sub-Saharan Africa. For proper risk assessment, the performance of some analytes needs additional improvement to achieve accurate quantitative data.

## Conclusion and outlook

In the present study, we report the development of a novel LC-MS/MS method for assessment of exposure to mycotoxins in stool during early life. To our knowledge, this is the first targeted LC-MS/MS method for human infant stool that includes several mycotoxin classes at once. The in-house validation revealed that 25 out of the 32 analytes fulfilled all stringent criteria despite the fact of covering a broad chemical space. It was successfully applied to a small set of samples from extremely premature infants from Austria and presumably highly exposed infants from Nigeria. The analytes which did not fulfill all the criteria (T2-toxin, AME, FB_1_, STC, OTA, NIV, CIT) can still be monitored, and this method can serve as a useful semi-quantitative screening tool for these mycotoxins. The co-occurrence of AME, FBs, and CIT in Nigerian samples reflects the current problem of mycotoxin exposure in the country and emphasizes the urgent need for large-scale cohort studies. It would be valuable to obtain data on mycotoxin levels in other types of matrices in order to do a full-exposure assessment and to better understand the toxicokinetic of a given mycotoxin. In addition, data from various regions with distinct climates would be of great value to explore the difference in exposure patterns.

## Declarations

The ethical approvals for the Austrian and Nigerian studies were obtained from the local ethics committee of the Medical University of Vienna (Ref. No.: 1348/2017) and from the Ethical Committee of Babcock University (Ref. No.: BUHREC585/18, BUHREC421/21R). For both studies, all parents gave written informed consent prior to study inclusion.
